# Dyslipidemia Patterns in Adults with Congenital Heart Disease: Focus on HDL Cholesterol

**DOI:** 10.3390/jcm14238357

**Published:** 2025-11-25

**Authors:** Efrén Martínez-Quintana, Fayna Rodríguez-González

**Affiliations:** 1 Cardiology Service, Complejo Hospitalario Universitario Insular-Materno Infantil, Avd. Marítima del Sur s/n, 35016 Las Palmas de Gran Canaria, Spain; 2 Department of Medical and Surgical Sciences, Faculty of Health Sciences, Universidad de Las Palmas de Gran Canaria, 35016 Las Palmas de Gran Canaria, Spain; 3 Hospital Universitario de Gran Canaria Dr. Negrín, 35010 Las Palmas de Gran Canaria, Spain

**Keywords:** congenital heart disease, dyslipidemia, low HDL cholesterol, arterial thrombosis, cardiovascular risk

## Abstract

**Introduction:** As survival in congenital heart disease (CHD) improves, identifying modifiable cardiovascular risk factors like dyslipidemia becomes increasingly important, though its features in adult CHD remain understudied. **Methods:** A retrospective study of 521 CHD patients (mean age 34 ± 14 years, 58% male) and 1782 matched controls (mean age 33 ± 11 years, 55% male) was conducted. Lipid profiles were assessed cross-sectionally at a single time point, and arterial thrombosis events were recorded over a mean follow-up of 5.8 years. **Results:** CHD patients had significantly lower total cholesterol (164.5 vs. 180.6 mg/dL), LDL (94.9 vs. 107.0 mg/dL), HDL (49.7 vs. 53.1 mg/dL), and triglycerides (97.0 vs. 102.4 mg/dL) compared to controls (all *p* < 0.05). Low HDL cholesterol (<40 mg/dL) was observed in 20% of CHD patients, nearly double the prevalence in the control group (11.6%; *p* < 0.001). This abnormality increased with CHD complexity: 15.2% in simple, 22.6% in moderate, and 28.9% in complex lesions. The proportion of patients with HDL < 40 mg/dL was higher in those with ventricular hypoplasia and Eisenmenger syndrome (*p* = 0.027). These groups also exhibited significantly higher NT-pro BNP levels, with a trend toward elevated C-reactive protein (CRP). Arterial thrombosis occurred in 4.0% of CHD patients versus 0.5% of controls (*p* < 0.001), with no significant differences between CHD subtypes. While overall lipid parameters did not differ significantly, the combination of low HDL and high LDL levels (mixed LDL + HDL pattern) was more common among patients with thrombosis (*p* = 0.005), although this association lost significance in binary logistic regression analysis. **Conclusions:** CHD patients exhibit a distinct lipid profile marked by lower HDL levels, particularly in complex cases and high-risk subtypes. Although the mixed low HDL/high LDL pattern was linked to thrombosis, this association was not maintained in multivariable analysis.

## 1. Introduction

Congenital heart disease (CHD) is the most common congenital malformation worldwide, affecting approximately 1% of live births [1]. Due to significant advancements in prenatal detection, surgical intervention, and chronic care, survival rates have markedly improved, resulting in a growing population of individuals with CHD living well into adulthood [2]. Consequently, CHD has transitioned from a predominantly pediatric condition to a lifelong chronic disease, often accompanied by an increasing burden of acquired cardiovascular risk factors. Among these, dyslipidemia has emerged as a central, modifiable contributor to atherosclerotic cardiovascular disease in the growing adult CHD population [3].

From an epidemiological standpoint, using clearly defined lipid cut-off values—such as total cholesterol ≥ 240 mg/dL or Low-Density Lipoprotein (LDL) cholesterol ≥ 130 mg/dL—offers more practical and clinically relevant information than relying solely on average lipid concentrations [4]. These cut-offs enable more precise identification of high-risk individuals, support standardized risk stratification, and improve the guidance of preventive measures across populations. This approach is especially important in patients with CHD, who display significant anatomical and physiological diversity. Factors such as abnormal cardiovascular physiology, chronic hemodynamic stress, systemic inflammation, and long-term pharmacotherapy can all influence lipid metabolism and alter atherosclerotic risk [5], making the use of standard risk prediction models and lipid management guidelines more challenging [6]. These complexities highlight the need for population-specific data to accurately assess cardiovascular risk and tailor preventive care for this unique group [7]. Consequently, adopting lipid thresholds specific to CHD subtypes, rather than applying general population standards, may enhance risk prediction and enable more effective, individualized management strategies.

This study aims to characterize dyslipidemia patterns in adults with CHD compared to controls, focusing on the distribution of lipid profiles by anatomical complexity and physiological classification. It also seeks to evaluate associations with demographic and clinical variables and to assess the relationship between lipid profiles and cardiovascular events in this population.

## 2. Methods

### 2.1. Study Design and Population

This retrospective study included patients with CHD followed at our specialized unit between January 2016 and January 2022. Inclusion criteria were age ≥18 years, a confirmed diagnosis of structural CHD established by echocardiography, cardiovascular magnetic resonance imaging, and/or cardiac catheterization, availability of complete clinical and laboratory data, and provision of written informed consent. Patients were excluded if they were under 18 years of age, had undergone any cardiac intervention (surgical or percutaneous) within the six months prior to inclusion, had an active malignancy, had a life expectancy of less than one year, or declined to provide informed consent. The control group comprised age- and sex-matched individuals recruited from community health centers within the same geographic region, attending for minor non-cardiac complaints.

### 2.2. Clinical Variables

In both groups, the age and sex were determined, and the presence of cardiovascular risk factors such as hypertension, diabetes mellitus, and smoking status was assessed. Additionally, ongoing treatment with statins, beta-blockers, diuretics, antiplatelets, or anticoagulant agents was evaluated. Oral anticoagulation included patients treated with acenocoumarol or direct oral anticoagulants. Body mass index (BMI) was calculated by dividing the patient’s weight in kilograms by the square of their height in meters (BMI = kg/m^2^). The diagnosis of atrial fibrillation was made through electrocardiographic evaluation. Patients with mechanical valve prostheses were identified through a comprehensive review of medical records. Cyanotic patients were defined as those with hemoglobin oxygen saturation levels below 90%. The incidence of arterial thrombosis during follow-up was also recorded. The study protocol was approved by the Institutional Ethics Committee and conducted following the principles of the Declaration of Helsinki. All participants provided written informed consent prior to inclusion in the study.

### 2.3. CHD Classification

In accordance with the classification proposed at the 32nd Bethesda Conference, cardiac defects were categorized as simple, moderate or severe complexity [8]. CHD were also classified into seven groups according to their main hemodynamic and functional effects at birth, before any intervention: (a) left-to-right shunts, producing volume overload without cyanosis (e.g., ventricular septal defect, atrial septal defect and patent ductus arteriosus); (b) obstructive lesions, associated with pressure overload (e.g., coarctation of the aorta, aortic or pulmonary stenosis); (c) cyanotic lesions with decreased pulmonary flow, characterized by reduced pulmonary circulation and cyanosis (e.g., tetralogy of Fallot, pulmonary or tricuspid atresia); (d) cyanotic lesions with parallel circulation, involving complete mixing of oxygenated and deoxygenated blood (e.g., transposition of the great arteries, truncus arteriosus); (e) ventricular hypoplasia, defined by single ventricle physiology and inadequate output (e.g., hypoplastic left heart syndrome, hypoplastic right ventricle, Fontan circulation); (f) valvular and atrioventricular defects, causing combined pressure and volume overload (e.g., complete atrioventricular canal defect, Ebstein anomaly); and (g) Eisenmenger syndrome, the result of irreversible pulmonary hypertension with shunt reversal and cyanosis. Finally, patients with CHD were additionally classified according to treatment history (no prior surgery, surgery during childhood, surgery during adulthood, or percutaneous intervention).

### 2.4. Laboratory Methods and Lipid Profile Definitions

Blood samples were collected after an overnight fast of at least 10 h and processed immediately. Glucose levels, hepatic enzymes—aspartate aminotransferase (AST) and alanine aminotransferase (ALT)—and lipid parameters, including total cholesterol, LDL cholesterol, high-density lipoprotein (HDL) cholesterol, and triglycerides, were quantified simultaneously using spectrophotometry on the Olympus AU 2700 analyzer. High-sensitivity C-reactive protein (hs-CRP) levels were measured using the immunoturbidimetric method on the same analyzer, while N-terminal pro-B-type natriuretic peptide (NT-pro-BNP) was assessed by immunoassay using the Siemens Stratus CS Acute Care Diagnostic System, exclusively in patients with CHD.

For clarity, we distinguish laboratory reference ranges from clinical cut-offs used to define dyslipidemia. Laboratory reference ranges (normal intervals for our analyzer) were total cholesterol 120–220 mg/dL, LDL cholesterol 0–155 mg/dL, HDL cholesterol 35–65 mg/dL, and triglycerides 30–200 mg/dL. In contrast, clinical dyslipidemia thresholds, based on ESC/ACC guidelines for cardiovascular risk management, were total cholesterol ≥ 240 mg/dL, LDL cholesterol ≥ 130 mg/dL, HDL cholesterol < 40 mg/dL, and triglycerides > 150 mg/dL [9,10].

Mixed dyslipidemia was defined as the presence of two abnormal lipid parameters. Specifically, either elevated LDL combined with elevated triglycerides (LDL + TG) or elevated LDL combined with low HDL (LDL + HDL). Although not explicitly defined in current guidelines, this approach is supported by prior studies recognizing the clinical relevance of combined lipid abnormalities in assessing cardiovascular risk [11].

### 2.5. Clinical Follow-Up

Clinical follow-up data in CHD patients were obtained from medical records or telephone interviews. Cardiovascular events were defined as follows: neurological events, including stroke and transient ischemic attack (TIA), were defined as acute or transient episodes of focal cerebral, spinal, or retinal dysfunction caused by ischemia; myocardial infarction was diagnosed when there was evidence of myocardial necrosis in a clinical context consistent with myocardial ischemia; and peripheral vascular disease included any disorder of the peripheral circulation requiring acute revascularization or hospitalization [12].

### 2.6. Statistical Analysis

Categorical variables were expressed as absolute numbers and percentages, and continuous variables were expressed as mean ± standard deviation (SD) or median [interquartile range], depending on their distribution. Comparisons between groups were performed using the chi-squared test or Fisher’s exact test for categorical variables and Student’s t-test or Mann–Whitney U test for continuous variables, as appropriate. To account for potential confounding factors, multivariate binary logistic regression analyses were performed to evaluate the independent association of clinical and biochemical variables with arterial thrombosis. Variables that were significant in univariate analyses (*p* < 0.05) were included in the multivariate models. Results are expressed as odds ratios (ORs) with 95% confidence intervals (CIs). A two-sided *p*-value < 0.05 was considered statistically significant. A post hoc power analysis based on the observed incidence of arterial thrombosis (4.0% in CHD patients vs. 0.5% in controls) indicated a statistical power of 99.9% (α = 0.05), confirming that the study sample was adequate to detect differences in this hard clinical outcome. All statistical analyses were conducted using SPSS Statistics version 24 (IBM Corp., Armonk, NY, USA).

## 3. Results

A total of 521 patients with CHD and 1782 control subjects were included in the analysis. There were no significant differences between groups in terms of age (34 ± 14 vs. 33 ± 11 years; *p* = 0.061) or sex distribution (male: 58% vs. 55%; *p* = 0.340). However, patients with CHD exhibited higher prevalences of hypertension (14% vs. 10%; *p* = 0.008) and diabetes mellitus (5% vs. 3%; *p* = 0.004), and a significantly greater proportion had a history of arterial thrombosis (4.0% vs. 0.5%; *p* < 0.001). Lipid profiles revealed significantly lower mean levels of total cholesterol (164.5 ± 39.2 vs. 180.6 ± 38.5 mg/dL; *p* < 0.001), LDL cholesterol (94.9 ± 32.1 vs. 107.0 ± 31.4 mg/dL; *p* < 0.001), HDL cholesterol (49.7 ± 11.7 vs. 53.1 ± 11.8 mg/dL; *p* < 0.001), and triglycerides (97.0 ± 53.1 vs. 102.4 ± 54.6 mg/dL; *p* = 0.045) in the CHD group. However, when stratified by clinical thresholds, only elevated LDL (≥130 mg/dL; *p* = 0.014) and low HDL (<40 mg/dL; *p* < 0.001) remained significantly different between groups (Table 1).

Regarding CHD complexity, 265 patients had simple lesions, 159 had moderate complexity, and 97 were classified as having severe complexity. Low HDL cholesterol (<40 mg/dL) was notably more frequent in CHD patients, increasing progressively from 15.2% in simple to 28.9% in severe complexity, compared to 11.6% in controls (*p* < 0.001). Similarly, patients with CHD had a significantly lower number of individuals with elevated total cholesterol levels (*p* = 0.047) and LDL cholesterol levels (*p* < 0.001) compared to those in the control group. On the contrary, no significant differences were observed in the number of patients with elevated triglyceride levels or mixed dyslipidemia patterns, either when comparing patients with CHD according to the complexity or when compared to the control population (Table 2).

CHD was also distributed according to anatomical subtypes into seven hemodynamic categories, with left-to-right shunts (n = 152) and obstructive lesions (n = 162) being the most frequent (Table 3). The most frequent lesions were ventricular septal defect (VSD, n = 86), aortic valve disease (n = 68), atrial septal defect (ASD, n = 60), pulmonary valve stenosis (n = 55), aortic coarctation (n = 47), repaired tetralogy of Fallot (TOF, n = 42), partial or complete atrioventricular septal defect (AVSD, n = 42), D-transposition of the great arteries (D-TGA, n = 24), congenitally corrected transposition of the great arteries (ccTGA, n = 14), double outlet right ventricle (DORV, n = 14), aortic stenosis (n = 11), single ventricle (n = 10), patent ductus arteriosus (PDA, n = 12), pulmonary atresia (n= 7), Ebstein anomaly (n = 6), tricuspid atresia (n = 4) and truncus arteriosus (n = 2) in decreasing order of prevalence. In addition, isolated cases of valvular prolapse or regurgitation, sub- and supravalvular aortic and pulmonary stenoses, systemic venous anomalies, and arteriovenous (AV) fistulas were also identified.

**Figure 1:**
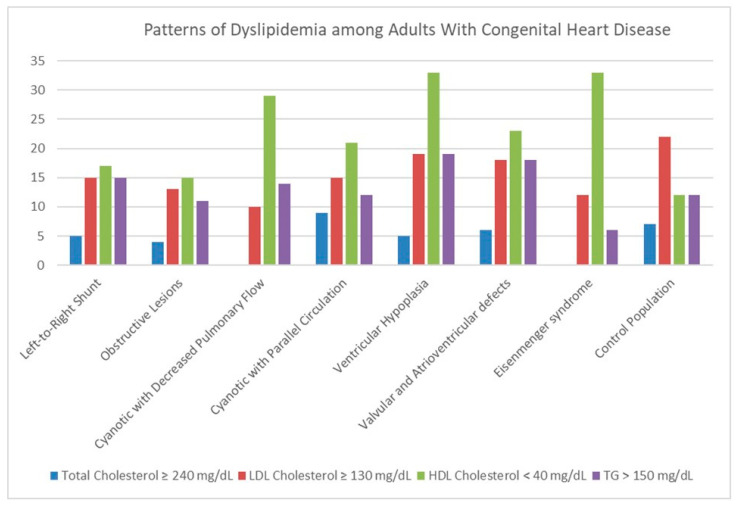
Patterns of dyslipidemia among adults with congenital heart disease and the control population. The figure shows subgroup-specific frequencies of total cholesterol ≥ 240 mg/dL (blue), LDL cholesterol ≥ 130 mg/dL (red), HDL cholesterol < 40 mg/dL (green), and triglycerides > 150 mg/dL (purple).

As shown in Table 3, most traditional cardiovascular risk factors and lipid abnormalities were evenly distributed across CHD anatomical subtypes, except for the proportion of patients with HDL < 40 mg/dL, which was higher in patients with ventricular hypoplasia and Eisenmenger syndrome (*p* = 0.027). In fact, both patient groups showed significantly higher levels of NT-pro BNP compared to the rest, and a trend towards elevated CRP levels was also observed. Meanwhile, arterial thrombosis rates remained low and did not differ significantly among subgroups. Figure 1 illustrates the lipid profile distribution across CHD subtypes and the control group, using a stacked bar chart to represent the proportion of each lipid abnormality. Low HDL cholesterol (<40 mg/dL) was significantly more common in patients with ventricular hypoplasia and Eisenmenger syndrome compared to controls (*p* < 0.001). In contrast, elevated LDL cholesterol (≥130 mg/dL) was less frequent in certain CHD subtypes—especially in patients with cyanosis and reduced pulmonary blood flow—compared to the control group (*p* = 0.014). There were no significant differences between CHD patients and controls in the prevalence of high total cholesterol, elevated triglycerides, or mixed lipid patterns, including mixed LDL + TG and mixed LDL + HDL abnormalities.

Regarding the type of intervention, 39% (n = 205) of patients had no surgical intervention, 49% (n = 253) underwent surgery during childhood, 4% (n = 22) had surgery in adulthood, and 8% (n = 41) received percutaneous treatment, reflecting the distribution within the total patient cohort. No significant differences were observed between these groups in terms of total cholesterol, LDL cholesterol, or triglyceride levels. However, low HDL cholesterol (<40 mg/dL) was significantly more frequent among patients with a history of childhood surgery (*p* = 0.008).

Among the 521 patients with CHD analyzed, 21 (4%) experienced arterial thrombosis, classified as cerebral, coronary, or peripheral. Cerebral thrombosis was the most common thrombotic event, occurring in 16 patients, followed by myocardial infarction in 4 patients and peripheral thrombosis in 1 patient. These events were observed over a mean follow-up period of 5.8 ± 2.3 years. Table 4 presents the clinical characteristics of CHD patients stratified by the presence of arterial thrombosis. Patients with thrombosis were significantly older (51.2 ± 14.1 vs. 33.9 ± 13.7 years; *p* < 0.001) and had higher prevalences of hypertension (*p* = 0.009), diabetes (*p* = 0.003), smoking history (*p* = 0.001), atrial fibrillation (*p* = 0.015), and cyanosis (*p* = 0.001). They were also more frequently treated with antiplatelet agents (*p* < 0.001), oral anticoagulants (*p* = 0.001), and statins (*p* < 0.001). While overall lipid parameters did not differ significantly, the combination of low HDL and high LDL levels (mixed LDL + HDL pattern) was more frequent among patients with thrombosis (*p* = 0.005), suggesting a potentially atherogenic profile in this subgroup.

Finally, as shown in Table 5, binary logistic regression analysis identified increasing age (adjusted OR 1.04; 95% CI: 1.01–1.08; *p* = 0.032), cyanosis (adjusted OR 6.81; 95% CI: 2.12–21.87; *p* = 0.001), and statin use (adjusted OR 4.31; 95% CI: 1.25–14.89; *p* = 0.021) as independent predictors of arterial thrombosis in CHD patients. While atrial fibrillation and mixed dyslipidemia (LDL + triglycerides) were significantly associated in univariate analyses, these associations did not remain statistically significant after multivariable adjustment, highlighting the multifactorial nature of thrombosis risk in this population.

## 4. Discussion

In our cohort of patients with CHD and matched controls, one of the most striking findings was the distinctive lipid profile observed in individuals with CHD. These patients exhibited significantly lower levels of total cholesterol, LDL cholesterol, HDL cholesterol, and triglycerides compared to controls, although no significant differences were found in statin use between the two groups. This pattern is consistent with previous systematic reviews and meta-analyses, which have reported significantly reduced lipid parameters in adults with CHD relative to the general population [13,14]. These differences may reflect a combination of chronic hemodynamic stress, nutritional or metabolic adaptations, lower body mass index, and possibly hepatic perfusion alterations or malabsorption in individuals with congenital heart defects [3]. 

In relation to different CHD subtypes, our study revealed distinct lipid profile patterns, highlighting the complex interplay between cardiac physiology and lipid metabolism. Notably, low HDL cholesterol (<40 mg/dL) was the most frequent lipid abnormality, especially prevalent in patients with ventricular hypoplasia and Eisenmenger syndrome, affecting roughly one-third of these groups.

This reduction in HDL concentrations observed in our CHD patients, especially in those with greater complexity, is likely driven by a combination of systemic inflammation, oxidative stress, and endothelial dysfunction—key features commonly present in the chronic conditions affecting young patients [15]. Inflammatory processes accelerate HDL catabolism, impair particle maturation, and downregulate pathways involved in HDL biosynthesis [16,17]. Oxidative stress promotes irreversible structural changes in HDL, making the particles more prone to clearance and loss of function [18]. Supporting this, a recent meta-analysis demonstrated elevated oxidative stress levels in patients with CHD, particularly those with cyanosis [19]. Additionally, endothelial dysfunction may further disrupt reverse cholesterol transport and impair HDL formation [20]. Furthermore, our CHD patients exhibited a trend toward higher hs-CRP levels, a marker of systemic inflammation, and statistically significantly higher NT-pro-BNP concentrations, a marker of heart failure, which may reflect an increased burden of cardiometabolic dysregulation. Similarly, HDL levels < 40 mg/dL were more frequent in patients who underwent surgery in early childhood, which may be attributed to the need for early surgical intervention in CHD cases with more pronounced structural and functional compromise.

Therefore, HDL cholesterol may hold prognostic relevance in adults with CHD, beyond its conventional role in lipid metabolism [21]. In this population, HDL cholesterol appears to be not only a marker of cardiovascular risk but also a potential biomarker reflecting the core pathological mechanisms driving complex CHD [22]. In fact, some authors have suggested that low HDL cholesterol levels are independently associated with adverse outcomes, including increased rates of hospitalization, thromboembolic events, and mortality in various clinical settings [23]. Given its pleiotropic functions—such as promoting cholesterol efflux, exerting antioxidant and anti-inflammatory effects, and supporting endothelial integrity—HDL cholesterol may serve as a more integrative biomarker of vascular health than traditional lipid measures [24,25].

Shifting to arterial thrombosis, our study confirmed a markedly higher prevalence in patients with CHD (4.0%) compared to controls (0.5%), reinforcing that CHD is a risk state for arterial vascular events. In retrospective analyses of CHD populations, arterial thrombotic events—particularly strokes and transient ischemic attacks—have been documented as more frequent relative to matched controls [26,27,28]. Importantly, differences in standard lipid abnormalities between patients with and without thrombosis were minimal, aside from certain mixed lipid patterns.

In the adjusted logistic regression analysis, age, cyanosis, and statin therapy emerged as independent predictors of arterial thrombosis. Age likely reflects cumulative vascular stress and comorbidities. Cyanosis highlights the prothrombotic environment of chronic hypoxemia—elevated viscosity, shear stress changes, endothelial activation, and coagulation disturbances [29]. The association between statin use and thrombosis likely reflects indication bias, as statins are typically prescribed to patients with higher vascular risk. In the general population, statins provide anti-inflammatory and antithrombotic benefits [30], so this association should not be interpreted as causal harm but rather as residual confounding or clinician-driven selection of higher-risk patients.

### Study Limitations

This study has several limitations. Its retrospective design introduces potential selection and information bias, and lipid values were obtained from a single measurement, preventing assessment of temporal trends or treatment effects. The number of arterial thrombotic events was low, increasing the risk of overfitting in multivariable analyses. Additionally, the control group was recruited from local outpatient clinics rather than the general population, which may limit comparability. We also did not adjust for multiple testing, raising the possibility of type I error. Finally, as a single-center study in a tertiary referral unit, generalizability may be limited, and the heterogeneity of adult CHD with relatively few cardiovascular events may have reduced our ability to detect certain associations. Despite these limitations, the study benefits from a well-characterized adult CHD cohort, matched controls, and adequate statistical power for major outcomes.

In summary, our findings highlight two key aspects: adults with CHD exhibit a distinct lipid profile—with globally lower cholesterol but a higher prevalence of low HDL, particularly in complex and cyanotic conditions—and they face an increased risk of arterial thrombosis that appears independent of traditional lipid abnormalities. Age, cyanosis, and statin use emerged as independent predictors, supporting a multifactorial thrombogenic environment in CHD. These results emphasize the need for CHD-specific risk stratification, closer surveillance, and future prospective studies to clarify mechanisms linking lipid metabolism, endothelial function, and hemostasis, as well as to evaluate targeted thromboprophylaxis. Longitudinal assessment of lipid profiles and the effects of lipid-lowering therapies should also be explored. Overall, integrating lipid evaluation into routine care may help refine risk reduction strategies in this vulnerable population.

## Figures and Tables

**Table 1:** Cardiovascular risk factors in CHD patients vs. controls.

Variable	CHD (n = 521)	Control (n = 1782)	*p*-Value
Age, years	34 ± 14	33 ± 11	0.061
Sex (male), n (%)	300 (57.6%)	984 (55.2%)	0.340
Hypertension, n (%)	73 (14%)	176 (9.9%)	0.008
Diabetes, n (%)	26 (5%)	45 (2.5%)	0.004
BMI, kg/m^2^	24 ± 5	24 ± 6	0.893
Smoker, n (%)	28 (5.4%)	290 (16.3%)	<0.001
Statins, n (%)	44 (8.7%)	122 (6.9%)	0.160
Arterial thrombosis, n (%)	21 (4%)	9 (0.5%)	<0.001
Dyslipidemia			
Total cholesterol, mg/dL	164.5 ± 39.2	180.6 ± 38.5	<0.001
Total cholesterol (≥240 mg/dL), n (%)	22 (4.2%)	133 (7.5%)	0.158
LDL cholesterol, mg/dL	94.9 ± 32.1	107.0 ± 31.4	<0.001
LDL cholesterol (≥130 mg/dL), n (%)	73 (14.0%)	392 (22.0%)	0.014
HDL cholesterol, mg/dL	49.7 ± 11.7	53.1 ± 11.8	<0.001
HDL (<40 mg/dL), n (%)	104 (20.0%)	207 (11.6%)	<0.001
TG, mg/dL	97.0 ± 53.1	102.4 ± 54.6	0.045
TG (≥150 mg/dL), n (%)	70 (13.5%)	270 (15.2%)	0.662
Mixed LDL + TG, n (%)	19 (3.7%)	93 (5.2%)	0.428
Mixed LDL + HDL, n (%)	9 (1.7%)	22 (1.2%)	0.808

CHD: Congenital Heart Disease; BMI: Body Mass Index; LDL: Low-Density Lipoprotein cholesterol; HDL: High-Density Lipoprotein cholesterol; TG: Triglycerides; Mixed LDL + TG: patients with both LDL ≥ 130 mg/dL and TG > 150 mg/dL; Mixed LDL + HDL: patients with both LDL ≥ 130 mg/dL and HDL < 40 mg/dL.

**Table 2:** Distribution of Cardiovascular Risk Factors Across Different Severity Levels of Coronary Heart Disease and the Control Group.

Variables	Simple CHD	Moderate CHD	Great CHD	Control Group	*p*-Value
Patients, n	265	159	97	1782	
Age, years	34 ± 15	33 ± 13	37 ± 14	33 ± 11	0.012
Sex (male), n (%)	157 (59.5%)	90 (56.6%)	53 (54.6%)	984 (55.2%)	0.657
BMI, kg/m^2^	24 ± 5	24 ± 5	24 ± 5	24 ± 6	0.897
Hypertension, n (%)	38 (14.4%)	28 (17.6%)	7 (7.2%)	176 (9.9%)	0.003
Diabetes, n (%)	16 (6.1%)	4 (2.5%)	6 (6.2%)	45 (2.5%)	0.005
Dyslipidemia					
Total Cholesterol ≥ 240, n (%)	14 (5.3%)	4 (2.5%)	4 (4.1%)	133 (7.5%)	0.047
LDL ≥ 130 mg/dL, n (%)	43 (16.3%)	17 (10.7%)	13 (13.4%)	392 (22.0%)	<0.001
HDL < 40 mg/dL, n (%)	40 (15.2%)	36 (22.6%)	28 (28.9%)	207 (11.6%)	<0.001
TG > 150 mg/dL, n (%)	39 (14.8%)	21 (13.2%)	11 (11.3%)	270 (15.2%)	0.702
Mixed LDL + TG, n (%)	9 (3.4%)	6 (3.8%)	4 (4.1%)	93 (5.2%)	0.524
Mixed LDL + HDL, n (%)	5 (1.9%)	2 (1.3%)	2 (2.1%)	22 (1.2%)	0.769

CHD: Congenital Heart Disease; BMI: Body Mass Index; LDL: Low-Density Lipoprotein cholesterol; HDL: High-Density Lipoprotein cholesterol; TG: Triglycerides; Mixed LDL + TG: patients with both LDL ≥ 130 mg/dL and TG > 150 mg/dL; Mixed LDL + HDL: patients with both LDL ≥ 130 mg/dL and HDL < 40 mg/dL.

**Table 3:** Demographic, clinical, and lipid profile characteristics of patients stratified by congenital heart disease subtype.

Variables	L-R Shunt	Obstructive	Cyanotic ↓ Pulm	Cyanotic Parallel	Ventricular Hypoplasia	Valv/Atriov Defects	Eisenmenger	*p*
**Patients, n (%)**	152 (29%)	162 (31%)	51 (10%)	34 (7%)	21 (4.0%)	68 (13%)	33 (6%)	
Age, years	33 ± 15	34 ± 14	35 ± 12	32 ± 12	34 ± 11	34 ± 13	46 ± 14	<0.001
BMI, kg/m^2^	24 ± 5	25 ± 5	24 ± 5	25 ± 4	24 ± 6	24 ± 5	24 ± 6	0.963
Male sex, n (%)	85 (56%)	100 (62%)	31 (61%)	23 (68%)	13 (62%)	36 (53%)	13 (39%)	0.201
HTN, n (%)	20 (13%)	31 (19%)	4 (8%)	4 (12%)	1 (5%)	10 (15%)	2 (6%)	0.192
Diabetes, n (%)	8 (5%)	8 (5%)	2 (4%)	0 (0%)	3 (14%)	1 (1%)	3 (9%)	0.174
Smoking, n (%)	8 (5%)	9 (6%)	6 (12%)	1 (3%)	0 (0%)	3 (4%)	1 (3%)	0.376
Atrial fibrillation, n (%)	10 (7)	4 (2)	4 (8)	1 (3)	3 (14)	2 (3)	9 (27)	<0.001
Arterial thrombosis, n (%)	9 (6%)	2 (1%)	4 (8%)	2 (6%)	1 (5%)	2 (3%)	1 (3%)	0.350
**Medical Treatment**								
Statin, n (%)	13 (9%)	11 (7%)	5 (10%)	2 (6%)	3 (14%)	7 (10%)	2 (6%)	0.886
Beta blocker, n (%)	13 (9%)	23 (15%)	8 (16%)	5 (15%)	9 (43%)	12 (18%)	9 (27%)	0.002
Diuretics, n (%)	13 (9%)	11 (7%)	9 (18%)	7 (21%)	10 (48%)	12 (18%)	15 (45%)	<0.001
Oral anticoagulant, n (%)	13 (9%)	19 (12%)	7 (14%)	6 (18%)	11 (52%)	6 (9%)	17 (52%)	<0.001
Antiplatelet, n (%)	11 (8%)	9 (6%)	10 (20%)	4 (12%)	4 (19%)	7 (10%)	8 (24%)	0.006
**Blood Test**								
Glucose, mg/dL	95 (89–99)	89 (89–101)	92 (87–99)	92 (87–101)	90 (84–96)	95 (81–101)	90 (84–106)	0.701
AST, U/L	22 (18–27)	21 (18–25)	22 (16–32)	23 (19–30)	23 (19–30)	22 (17–26)	25 (19–31)	0.096
ALT, U/L	16 (13–22)	17 (13–22)	18 (14–34)	20 (16–29)	21 (12–30)	17 (13–32)	18 (13–27)	0.788
Hs-CRP, mg/L	0.2 (0.06–0.45)	0.1 (0.05–0.4)	0.1 (0.05–0.3)	0.1 (0.07–0.4)	0.2 (0.1–0.4)	0.1 (0.06–0.5)	0.5 (0.1–1.4)	0.059
NT-pro-BNP, pg/mL	38 (13–80)	41 (9–101)	129 (48–250)	105 (65–312)	294 (97–1114)	80 (24–176)	426 (164–999)	<0.001
**Dyslipidemia**								
Total Chol ≥ 240 mg/dL, n (%)	7 (5%)	7 (4%)	0 (0%)	3 (9%)	1 (5%)	4 (6%)	0 (0%)	0.421
LDL ≥ 130 mg/dL, n (%)	22 (15%)	21 (13%)	5 (10%)	5 (15%)	4 (19%)	12 (18%)	4 (12%)	0.897
HDL < 40 mg/dL, n (%)	26 (17%)	22 (14%)	15 (29%)	7 (21%)	7 (33%)	16 (24%)	11 (33%)	0.027
TG > 150 mg/dL, n (%)	23 (15%)	18 (11%)	7 (14%)	4 (12%)	4 (19%)	12 (18%)	2 (6%)	0.623
Mixed LDL + TG, n (%)	6 (4%)	3 (2%)	2 (4%)	3 (9%)	1 (5%)	4 (6%)	0 (0%)	0.378
Mixed LDL + HDL, n (%)	3 (2%)	2 (1%)	1 (2%)	1 (3%)	1 (5%)	1 (1%)	0 (0%)	0.885

HTN: hypertension, Diabetes: diabetes mellitus, AST: Aspartate Aminotransferase, ALT: Alanine Aminotransferase, Hs-CRP: high-sensitivity C-Reactive Protein, NT-pro-BNP: N-terminal pro b-type Natriuretic Peptide, LDL: Low-Density Lipoprotein, HDL: High-Density Lipoprotein, TG: Triglycerides, Mixed LDL + TG: mixed dyslipidemia with elevated LDL and triglycerides, Mixed LDL + HDL: mixed dyslipidemia with elevated LDL and low HDL. L-R Shunt (Left-to-Right Shunt), Obstructive (Obstructive Lesions), Cyanotic ↓ Pulm (Cyanotic with Decreased Pulmonary Flow), Cyanotic Parallel (Cyanotic with Parallel Circulation), Ventricular Hypoplasia (single ventricle physiology), Valv/Atriov Defects (Valvular and Atrioventricular Defects), and Eisenmenger (Eisenmenger Syndrome).

**Table 4:** Clinical characteristics according to the presence of arterial thrombosis in patients with congenital heart disease.

Variables	Arterial Thrombosis		*p*-Value
	No	Yes	
Patients, n	500	21	
Age, years	33.9 ± 13.7	51.2 ± 14.1	<0.001
BMI, kg/m^2^	24.3 ± 5.2	26.3 ± 4.9	0.128
Sex (male), n (%)	288 (57.6%)	12 (57.1%)	0.967
Hypertension, n (%)	66 (13.2%)	7 (33.3%)	0.009
Diabetes, n (%)	22 (4.4%)	4 (19.0%)	0.003
Smoker and ex-smoker, n (%)	36 (7.2%)	4 (19.0%)	0.001
Atrial fibrillation, n (%)	29 (5.8%)	4 (19.0%)	0.015
Mechanical valve prosthesis, n (%)	22 (4.4%)	0 (0.0%)	0.255
Cyanosis, n (%)	38 (7.6%)	6 (28.6%)	0.001
Antiaggregation, n (%)	43 (8.6%)	10 (47.6%)	<0.001
Oral anticoagulation, n (%)	71 (14.2%)	9 (42.9%)	0.001
Statins, n (%)	35 (7.0%)	9 (42.9%)	<0.001
Dyslipidemia			
Total Chol ≥ 240, n (%)	21 (4.2%)	1 (4.8%)	0.900
LDL ≥ 130 mg/dL, n (%)	70 (14.0%)	3 (14.3%)	0.971
HDL < 40 mg/dL, n (%)	99 (19.8%)	2 (9.5%)	0.625
TG > 150 mg/dL, n (%)	68 (13.6%)	2 (9.5%)	0.929
Mixed LDL + TG, n (%)	19 (3.8%)	0 (0.0%)	0.363
Mixed LDL + HDL, n (%)	7 (1.4%)	2 (9.5%)	0.005

CHD: Congenital Heart Disease; BMI: Body Mass Index; LDL: Low-Density Lipoprotein cholesterol; HDL: High-Density Lipoprotein cholesterol; TG: Triglycerides; Mixed LDL + TG: patients with both LDL ≥ 130 mg/dL and TG > 150 mg/dL; Mixed LDL + HDL: patients with both LDL ≥ 130 mg/dL and HDL < 40 mg/dL.

**Table 5:** Results of the binary logistic regression analyses of congenital heart disease patients with and without arterial thrombosis.

Covariates	OR (Crude) (95% CI)	*p*	OR (Adjusted) (95% CI)	*p*
Age, years	1.06 (1.04–1.08)	<0.001	1.04 (1.01–1.08)	0.032
Hypertension, n (%)	3.29 (1.28–8.45)	0.013	0.98 (0.22–2.94)	0.745
Diabetes, n (%)	5.11 (1.59–16.47)	0.006	2.18 (0.54–8.83)	0.273
Smoker, n (%)	2.61 (1.29–5.25)	0.007	1.91 (0.84–4.34)	0.124
Atrial fibrillation, n (%)	3.81 (1.20–12.07)	0.023	1.14 (0.27–4.75)	0.853
Cyanosis, n (%)	4.86 (1.71–13.26)	0.002	6.81 (2.12–21.87)	<0.001
Statins, n (%)	10.52 (4.08–27.08)	<0.001	4.31 (1.25–14.89)	0.021
Mixed LDL + HDL, n (%)	7.41 (1.44–38.10)	0.016	4.45 (0.75–26.31)	0.100

Mixed LDL + HDL: patients with both LDL ≥ 130 mg/dL and HDL < 40 mg/dL, OR: Odds Ratio, CI: Confidence Interval. Adjusted ORs were obtained from a multivariate binary logistic regression model including variables significant in univariate analyses (*p* < 0.05).

## Data Availability

Due to the sensitive nature of the patient data and to ensure confidentiality, the datasets generated and analyzed during the current study are not publicly available.
